# Age exacerbates the CCR2/5-mediated neuroinflammatory response to traumatic brain injury

**DOI:** 10.1186/s12974-016-0547-1

**Published:** 2016-04-18

**Authors:** Josh M. Morganti, Lara-Kirstie Riparip, Austin Chou, Sharon Liu, Nalin Gupta, Susanna Rosi

**Affiliations:** Brain and Spinal Injury Center, University of California, 1001 Potrero Ave, Bldg. 1, Room 101, San Francisco, CA 94110 USA; Department of Physical Therapy and Rehabilitation Science, University of California, San Francisco, CA USA; Neuroscience Graduate Program, University of California, San Francisco, CA USA; Department of Neurological Surgery, University of California, San Francisco, CA USA; Department of Pediatrics, University of California, San Francisco, CA USA

**Keywords:** Microglia, Macrophage, CCR2, Chemokine, Antagonist, Aging, Neurotrauma

## Abstract

**Background:**

Traumatic brain injury (TBI) is a major risk factor for the development of multiple neurodegenerative diseases, including Alzheimer’s disease (AD) and numerous recent reports document the development of dementia after TBI. Age is a significant factor in both the risk of and the incidence of acquired brain injury. TBI-induced inflammatory response is associated with activation of brain resident microglia and accumulation of infiltrating monocytes, which plays a pivotal role in chronic neurodegeneration and loss of neurological function after TBI. Despite the extensive clinical evidence implicating neuroinflammation with the TBI-related sequelae, the specific role of these different myeloid cells and the influence of age on TBI-initiated innate immune response remain unknown and poorly studied.

**Methods:**

We used gene profiling and pathway analysis to define the effect of age on inflammatory response at the time of injury. The recruitment of peripheral CCR2^+^ macrophages was delineated using the *CX3CR1*^*GFP/+*^*CCR2*^*RFP/+*^ reporter mouse. These responses were examined in the context of CCR2/5 antagonism using cenicriviroc.

**Results:**

Unsupervised gene clustering and pathway analysis revealed that age predisposes exacerbated inflammatory response related to the recruitment and activation of peripheral monocytes to the injured brain. Using a unique reporter animal model able to discriminate resident versus peripherally derived myeloid cells, we demonstrate that in the aged brain, there is an increased accumulation of peripherally derived CCR2^+^ macrophages after TBI compared to young animals. Exaggerated recruitment of this population of cells was associated with an augmented inflammatory response in the aged TBI animals. Targeting this cellular response with cenicriviroc, a dual CCR2/5 antagonist, significantly ameliorated injury-induced sequelae in the aged TBI animals.

**Conclusions:**

Importantly, these findings demonstrate that peripheral monocytes play a non-redundant and contributing role to the etiology of trauma-induced inflammatory sequelae in the aged brain.

## Background

Traumatic brain injury (TBI) is an environmental risk factor for the development of many neurological disorders including degenerative diseases such as Alzheimer’s and early onset dementia [1, 2]. Age significantly increases both the risk and incidence of acquired brain injury [3]. Elderly individuals are particularly vulnerable to TBI and have clinically worse outcomes after TBI with increased morbidity and mortality, and reduced functional recovery [4, 5]. While age as a prognostic factor after TBI has long been recognized [6], limited attention has been devoted to understanding and modulating the pathologic processes that contribute to poor outcomes seen in aged individuals.

Brain injury initiates an immediate response involving multiple cellular effectors of the innate immune response, notably the brain’s resident tissue macrophage and microglia, as well as the recruitment of peripherally derived monocytes/macrophages presumably through the disruption of the blood-brain barrier [7–9]. Aging in humans and rodents has been shown to alter this response [10, 11]. In the current study, we used unsupervised gene clustering and pathway analysis to define the effect of age on injury-induced inflammatory sequelae. Our data show that age predisposes the injured brain to an exaggerated inflammatory response involving the enrichment of chemotactic mediators associated with the recruitment and activation of the peripheral monocytes. Specifically, our data show that aged TBI animals have an exacerbated response in the production of ligands that bind to both CCR2 and CCR5 at a critical time point associated with migration of peripheral monocytes/macrophages to the injured brain parenchyma. This molecular response was paralleled by an increased recruitment of CCR2^+^ macrophages to the injured brain of aged mice. Given the exacerbated CCR2/5 cellular and molecular signatures, we used a novel small molecule antagonist for CCR2/5 to abrogate this phenotype. Using a clinically relevant pharmacological approach, our data show that the age-related phenotype in response to injury is significantly mitigated in treated animals. These data implicate a new important role for the accumulation of monocyte/macrophages in the maintenance of neuroinflammatory sequelae following neurotrauma in the aged brain.

## Methods

### Animals

All experiments were conducted in accordance with the National Institutes of Health Guide for the Care and Use of Laboratory Animals and were approved by the Institutional Animal Care and Use Committee of the University of California (San Francisco, CA). Adult 3- (young) and 23-month-old (aged) male and female *CX3CR1*^*GFP/+*^*CCR2*^*RFP/+*^ (double heterozygous (Dbl-Het)) and *C57BL6/J* (wild type (WT)) male mice were used for all experiments. Dbl-Het mice (*n* = 16) were generated as previously described [12] and genotyped using a commercial service (Transnetyx), while WT mice (*n* = 48) were purchased from the National Institute on Aging animal colony. Mice were group housed in environmentally controlled conditions with reverse light cycle (12:12 h light:dark cycle at 21 ± 1 °C) and provided food and water ad libitum.

### Surgical procedure

All animals were randomly assigned and divided as equally possible between sexes (Dbl-Het) to their treatment group. Animals were anesthetized and maintained with 2.5 % isoflurane with a non-rebreathing nose cone and passive exhaust system connected to a stereotaxic frame (David Kopf). Once animals were secured with non-traumatic ear bars, eye ointment was applied and their heads were cleared of any hair around the scalp. Following betadine application, a midline incision was made through the scalp. TBI was reproduced using the controlled cortical impact model in the parietal lobe as previously described [13]. A craniectomy was created using an electric microdrill with center point to the coordinates: 2.0 mm; mediolateral, 2.0 mm, with respect to the bregma. Explicit attention was paid to prevent damage to the dura during craniectomy; any animal in which the dura was disrupted, as assessed by excessive bleeding, was omitted from the study and replaced by another littermate. After craniectomy, contusion was achieved using a 3.0-mm convex tip attached to an electromagnetic impactor (Leica) mounted to the digitally calibrated manipulator arm. To impact flush with the natural curvature of the head/tissue, the manipulator arm was rotated 20° on the vertical axis. The parameters for impact were for a contusion depth of 0.95 mm (from dura), velocity was constant at 4.0 m/s, and the impact was sustained for of 300 ms. After CCI injury, the scalp was sutured and each animal received 0.5 ml of physiologic saline (i.p.) before being placed in a water-heated incubation chamber (37 °C) until they fully recovered as exhibited by resumption of movement and grooming. Sham animals were treated to the above parameters, except that the CCI injury was omitted. All animals fully recovered from surgical procedures and exhibited normal weight gain for the duration.

### Tissue collection

All mice were euthanized using a mixture of ketamine (150 mg/kg)/xylazine (15 mg/kg) in accordance with standard animal protocols. For flow cytometry endpoints; once the animal was completely anesthetized, the chest cavity was opened and transcardially perfused using ice-cold Hank’s balanced salt solution without calcium and magnesium (HBSS; Gibco). Immediately after perfusion, mice were decapitated and the ipsilateral brain hemisphere was placed into ice-cold RPMI-1640 medium without phenol (RPMI; Gibco). For qRT-PCR endpoints, animals were rapidly killed via cervical dislocation. Each brain was quickly removed, and the ipsilateral hippocampi were dissected and snap frozen.

### Cenicriviroc administration

Cenicriviroc (CVC; Tobira Therapeutics), a small molecule dual antagonist for the human orthologs of CCR2 and CCR5 was dissolved in solution of 0.5 % hydroxypropyl methylcellulose (vehicle; HPMC + 1.0 % Tween80) to 5 mg/mL. Aged TBI animals were randomly divided between two treatment groups; vehicle and CVC. Two hours following surgery, animals were dosed at 100 mg/kg BID via oral gavage at 2 and 10 h post-surgery intervals. This treatment strategy was chosen to provide sufficient coverage of CCR2/5 through 24 h, with peak plasma concentrations of CVC reached by hour 2 (data not shown).

### Flow cytometry

Brain hemispheres in RMPI were used for leukocyte isolation following standard procedures [14]. Fc receptor blocking was performed before all staining procedures using an anti-CD16/32 antibody (BD Pharmigen). The following reagents were used for labeling isolated macrophages: ZombieAqua (BD Biolegend), CD11b Alexafluor 700 (BD Pharmigen), and F4/80 APC (Invitrogen) CD45 FITC (AbD Serotec) Ly6C PE (AbD Serotec). Mandibular blood draws from naïve *CCR2*^*RFP/RFP*^ and *CX3CR1*^*GFP/GFP*^ mice were used as positive controls for RFP and GFP expression, respectively. Additionally, naïve WT isolated leukocytes served as negative control for RFP and GFP expression. Spectral compensation was achieved using polystyrene microparticles (BD Pharmigen) in combination with each of the above listed conjugated antibodies following manufacturer’s suggested protocol. Standard staining procedures were conducted as previously described [14] before analysis on FACSAria III cell sorter (BD Biosciences). Gating parameters for both WT and Dbl-Het endpoints were used as previously described [13]. All samples were run in duplicate. Flow cytometric data were analyzed using FlowJo (Treestar; v9.9).

### qRT-PCR

Dissected ipsilateral hippocampi were used for all gene expression analyses. RNA isolation and cDNA conversion were completed as previously described [13]. RNA concentration and quality were determined using a NanoDrop (Thermo Scientific). Three hundred nanograms of RNA was reverse transcribed using High-Capacity cDNA Reverse Transcription Kit (Applied Biosystems). For inflammatory profiling arrays [15], (Qiagen, #330131) equal volumes of cDNA for each sample were pooled (*n* = 8 animals/group/pool) and run on a single plate per condition (e.g., young sham, young TBI, aged sham, and aged TBI); cycling conditions were followed as suggested by manufacturer. Select analytes from the profiling arrays were validated using individual samples (*n* = 8/group) carried out in duplicate using SYBR Green Master Mix (Applied Biosystems) following manufacturer’s suggested protocol. The relative expression of target genes was determined by the 2^−ΔΔCt^ method and normalized against beta-actin gene expression using a Statagene Mx3005P Real-Time PCR system. Specifically, the multiple genes were analyzed using the following primer sequences (5′ to 3′ sense/antisense);

*CCL2* (GCTGACCCCAAGAAGGAATG/GTGCTTGAGGTGGTTGTGGA), *CCL8* (GGGTGCTGAAAAGCTACGAGAG/GGATCTCCATGTACTCACTGACC), *CCL7* (CAGAAGGATCACCAGTAGTCGG/ATAGCCTCCTCGACCCACTTCT), *CCL12* (CAGTCCTCAGGTATTGGCTGGA/TCCTTGGGGTCAGCACAGAT), *CCL5* (CCTGCTGCTTTGCCTACCTCTC/ACACACTTGGCGGTTCCTTCGA), *TNFα* (TGCCTATGTCTCAGCCTCTTC/GAGGCCATTTGGGAACTTCT), *IL-1β* (TGTAATGAAAGACGGCACACC/TCTTCTTTGGGTATTGCTTGG), *Arg1* (GAACACGGCAGTGGCTTTAAC/TGCTTAGCTCTGTCTGCTTTGC), *CD36* (GGACATTGAGATTCTTTTCCTCTG/GCAAAGGCATTGGCTGGAAGAAC), *CX3CL1* (GGCTAAGCCTCAGAGCATTG/CTGTAGTGGAGGGGGACTCA), *CXCL2* (CATCCAGAGCTTGAGTGTGACG/GGCTTCAGGGTCAAGGCAAACT), *PTX3* (CGAAATAGACAATGGACTTCATCC/CATCTGCGAGTTCTCCAGCATG), JAK2 (GCTACCAGATGGAAACTGTGCG/GCCTCTGTAATGTTGGTGAGATC), JMJD3 (AGACCTCACCATCAGCCACTGT/TCTTGGGTTTCACAGACTGGGC), CD86 (ACGTATTGGAAGGAGATTACAGCT/TCTGTCAGCGTTACTATCCCGC), CD163 (GGCTAGACGAAGTCATCTGCAC/CTTCGTTGGTCAGCCTCAGAGA), *gp91*^*phox*^ (ACTCCTTGGGTCAGCACTGG/GTTCCTGTCCAGTTGTCTTCG)*, p22*^*phox*^ (GCTCATCTGTCTGCTGGAGTATC/CGGACGTAGTAATTCCTGGTGAG), *p40*^*phox*^ (CAAAGACCTGCTAGCGCTCATG/CCACATCCTCATCTGACAGCAG)*, p47*^*phox*^ (GCTGACTACGAGAAGAGTTCGG/CCTCGCTTTGTCTTCATCTGGC)*, p67*^*phox*^ (GCAGAAGAGCAGTTGGCATTGG/CTGCCTCTCATTTGGACGGAAC). All primer pairs were independently validated using a standard curve of serially diluted mouse cDNA before use in any endpoint. In each PCR analysis, template and RT controls were included to account for contamination. Gene expression data are represented as the Log_2_ fold change relative to young sham or to aged TBI-vehicle (for CVC endpoints).

### Hierarchical clustering analysis

Multi Experiment Viewer (v4.8) was used for hierarchical cluster analysis. This was performed using Pearson’s correlation for distance measure algorithm with average linking clustering parameters to identify multiple samples with similar expression patterns.

### Ingenuity Pathway Analysis (IPA)

Data from mini arrays were analyzed using IPA. Differentially expressed genes (young TBI, aged sham, and aged TBI versus young sham) were uploaded into IPA and refined to limit only expression values of **±**1.5-fold change before core analyses were commenced. Core analyses of the three groups were analyzed by comparison for the diseases and biological function analyses. These functional analyses were sorted by activation *z*-score of aged TBI group in a descending list, with exogenous chemicals omitted.

### Statistical analyses

All analyses were performed in Prism v6.0 (GraphPad) using Student’s *t* test and one-way ANOVA with Tukey HSD correction for multiple comparisons for both flow cytometry and gene expression analyses. Significance for all measures was assessed at *p* < .05.

## Results

### Age alters gene expression profiles as a response to injury

**Fig. 1:**
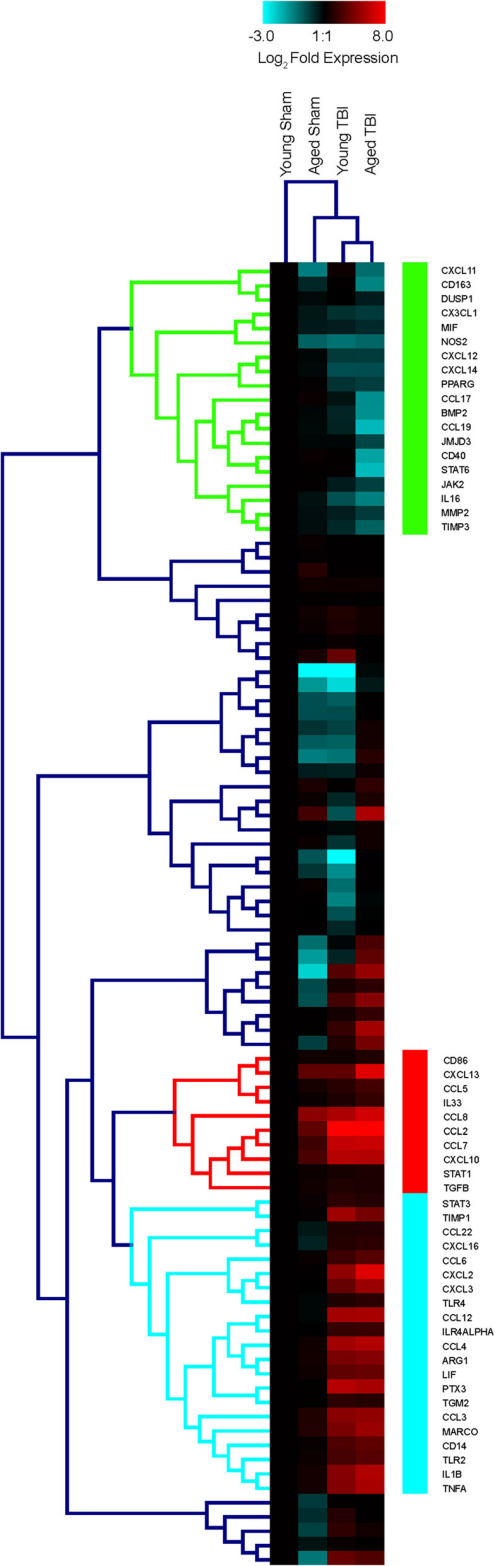
Inflammatory profiling of the TBI brain. Ipsilateral hippocampi pooled from sham and injured animals (*n* = 8/group) of 3-month (young; Y) and 23-month (aged; A) 24 h after surgery for gene array analysis. Inflammatory profiling array revealed clusters of enrichment and downregulation of genes across three groups; young TBI, aged sham, and aged TBI, relative to young sham expression levels, all data were Log_2_ transformed. Three specific clusters were examined, wherein the genes within the green cluster were downregulated in the aged TBI group, the *red gene cluster* showed marked enrichment for aged TBI, and lastly the *aquamarine cluster* showed similar enrichment of genes as a response to TBI, regardless of age. In heatmap; *teal* downregulated, *red* upregulated

We first conducted gene profiling of the injured parenchyma to examine the effect of age upon neuroinflammatory response to injury. Unsupervised hierarchical clustering (Fig. 1) revealed unique arrangements of both enriched and downregulated responses as a result of injury and/or age. Of these expression clusters, we examined three that visually represented alignment of genes that were downregulated in the aged TBI group (Fig. 1; green), upregulated in the aged TBI group (Fig. 1; red), and upregulated as a result of injury (Fig. 1; aquamarine). In general, the downregulated genes of the green cluster represented a repressed inflammatory function as a combination of age and injury. By contrast, the red cluster linked a group of genes related to recruitment and activation of pro-inflammatory monocytes. Notably, within this expression cluster, our data show increased responses of several CCR2 and CCR5 cognate ligands (e.g., CCL2, CCL7, CCL8, CCL5). While the aquamarine (Fig. 1) cluster was distinct from the changes found within the red cluster, there was a similar activation profile found between both TBI groups hierarchically. There was a heterogeneous mix of chemokines, cytokines, and signal transducers, and metabolic mediators found throughout this gene cluster, which seem to be indicative of a general inflammatory response to brain injury, as there was little to no activation of these mediators in the aged sham group (Fig. 1).

**Fig. 2:**
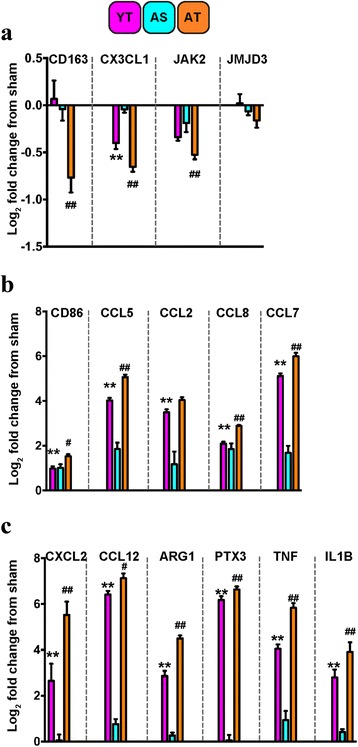
Age exacerbates inflammatory gene signatures after TBI. Overall, the aged TBI group had exacerbated responses, whether up- or downregulated, to TBI when compared to their young TBI counterparts. Select genes from each of the three clusters were validated using *n* = 8/group. **a** Genes from the “*green*” hierarchical cluster aligned with pooled responses observed in the array. Specifically, detailing that age significantly affected, via downregulation, gene expression of CD163, CX3CL1, and JAK2, but not JMJD3, which had only a visual trend. **b** Genes selected from the “*red*” cluster for analysis showed marked upregulation of CD86, CCL5, CCL8, and CCL7 in the aged TBI group. **c** Similarly, genes from the “*aquamarine*” hierarchical cluster all displayed significant upregulation in expression response as a function of age, compared to young TBI. *YT* young TBI; *magenta*, *AS* aged sham; *aquamarine*, *AT* aged TBI; *orange*. Young sham expression values are equivalent to zero. Data were analyzed using two-way ANOVA with Tukey’s PSD for multiple comparisons and presented as mean + SEM. *p < 0.05, **p < 0.01 comparing young TBI to young sham. ^#^ *p* < 0.05, ^##^ *p* < 0.01 comparing aged TBI to young TBI

Selected genes were validated within each of the green, red, and aquamarine clusters of interest. In line with the pooled observations from the array and cluster, four genes from the green cluster (Fig. 1): CD163, CX3CL1, JAK2, and JMJD3 were disproportionately downregulated in the aged TBI group, compared to young TBI (Fig. 2a). Potentially indicating that as a result of age, there is a maladaptive response to injury related to the restraint of macrophage activation, similar to what is observed in humans [16]. Interestingly, we show that the anti-inflammatory [17] chemokine CX3CL1 is significantly downregulated in the aged TBI group compared to young animals following TBI. These findings are similar to a report on brain aging in healthy rodents that showed CX3CL1 expression is decreased with age [18], which can lead to pro-inflammatory neurotoxic responses [19, 20].

Comparatively, when we quantified selected genes from the red cluster (Fig. 1), we found that compared to young TBI, the aged TBI group had increased expression of CD86, a marker linked specifically with microglia [21], as well as CCL8, CCL7, and CCL5, which are ligands for CCR2 and/or CCR5 (Fig. 2b). However, we did not observe an increase of CCL2 gene expression in the aged TBI group, relative to young TBI as has been previously reported [22]. Although CCL2 is the strongest chemoattractant for CCR2^+^ monocytes/macrophages, the increased presence of other constituent ligands (e.g., CCL8, CCL7, CCL5) may indicate an additive effect. As a result of age, the increased presence of multiple monocyte chemotactic mediators would allow a greater permissive environment for increased recruitment.

Similarly, validation of selected genes within the aquamarine cluster (Fig. 1) showed that there was an exacerbated response to injury due to age (Fig. 2c). Specifically, as a response to injury, aged TBI animals had significantly increased expression of the inflammatory chemokines CXCL12, and CCL12, and similarly with pro-inflammatory cytokines TNFα and IL-1β, as well as PTX3. Arg1 was also significantly induced relative to young TBI. The elevated responses we observed are analogous to gene signatures from isolated CNS monocytes/macrophages in a mouse model of EAE-induced neuroinflammation [23].

### Age alters injury-induced putative upstream and downstream regulatory networks

**Fig. 3:**
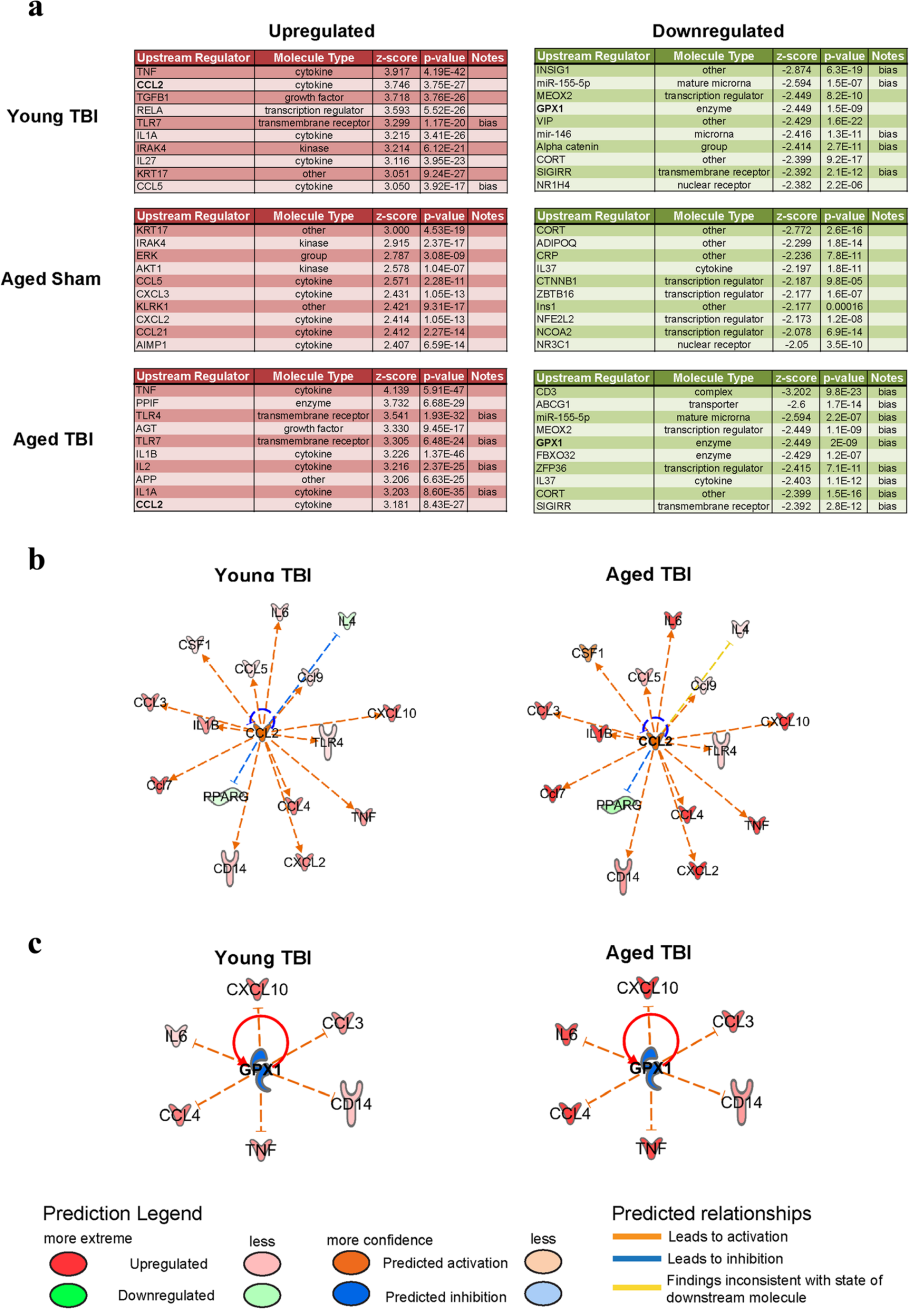
IPA upstream analysis displays heterogeneous regulatory components associated with inflammatory response. Gene array data were loaded into IPA software; genes with a fold change (relative to young sham) ≥1.5 or ≤−1.5 were included for upstream regulator analysis. **a** Upregulated and downregulated molecules were sorted via their respective activation *z*-score and the top ten regulators (up and down) are presented for each condition. **b**, **c** Representative regulatory networks for putative upstream mediators associated with young TBI and aged TBI, with CCL2 representing an upregulated response and GPX1 representing a downregulated response. Both CCL2 and GPX1 show dissimilar expression responses for young TBI versus aged TBI

Next, we examined the gene expression profiles for translational observations through identification of putative upstream and downstream functional analyses using IPA software. This software converts gene expression data into recognized functional matrices related to disease etiology. Using this approach, IPA software identified regulatory genes associated with predicted upstream networks (Fig. 3a). Of these putative regulators, we sorted these responses by descending activation *z*-score and selected the top ten regulators that were either up- or downregulated for each of the three conditions (e.g., young TBI, aged sham, aged TBI), relative to young sham animals. These analyses revealed heterogeneous regulatory responses with respect to the variety of genes identified; however, there were a few instances where these regulators were conserved between conditions. Interestingly, among the conserved regulators between both young and aged TBI groups were the activation of CCL2 (Fig. 3b) and downregulation of GPX1 (Fig. 3c), involved in the recruitment of CCR2^+^ leukocytes and regulation of oxidative stress, respectively.

**Fig. 4:**
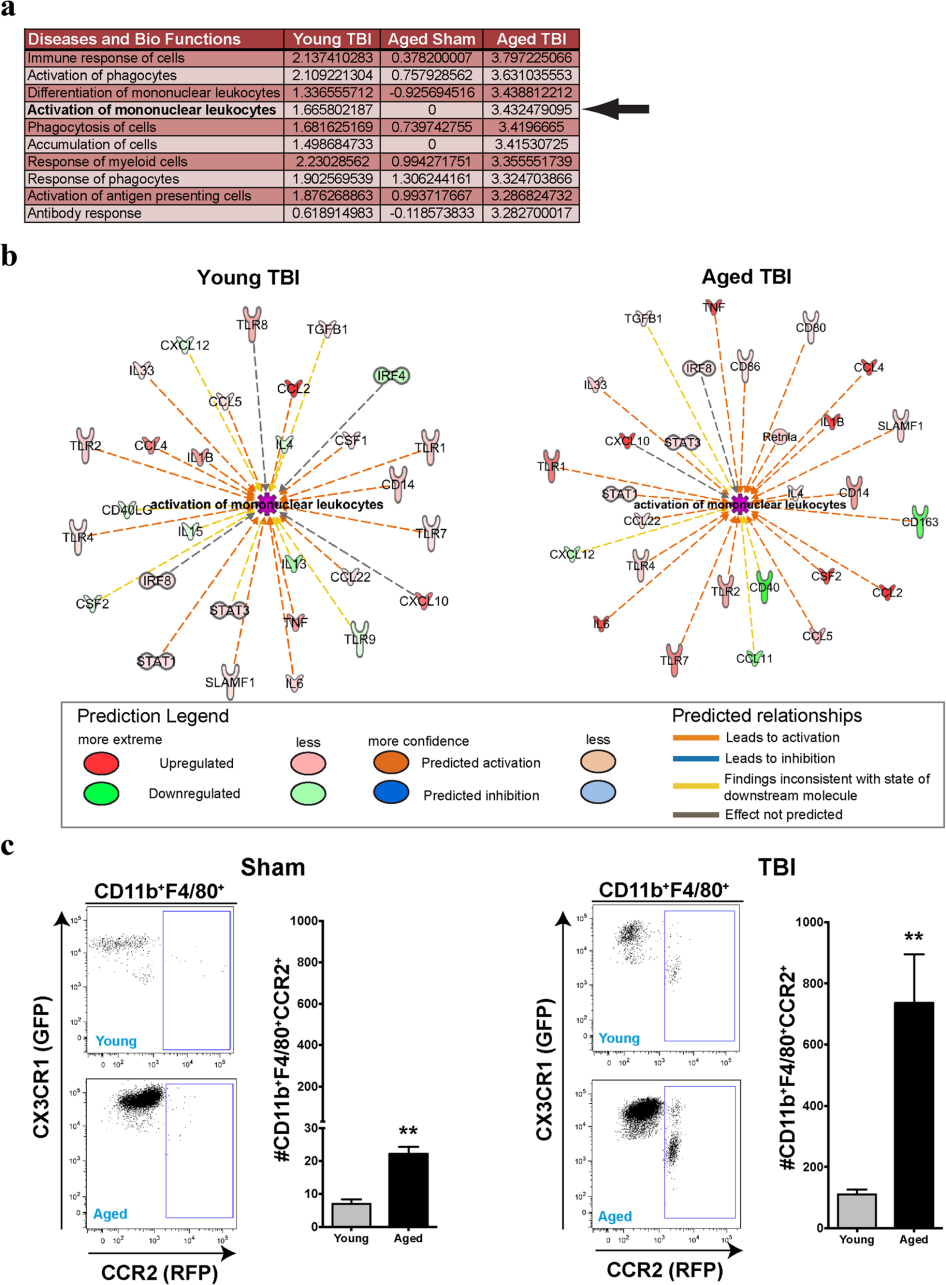
Gene profiling predicts exaggerated activation and recruitment of monocytes in aged TBI. Gene array data were uploaded into IPA software, genes with a fold change (relative to young sham) ≥1.5 or ≤−1.5 were included for disease and biological function analysis. **a** Upregulated functions across all three groups were sorted by activation *z*-score of the aged TBI group, only the top ten functions are presented with the *black arrow* emphasizing the activation of monocytes. **b** Functional network diagram of predicted and measured regulators is presented for both young TBI and aged TBI, which highlight an overrepresented activation for this function for aged TBI group. **c** Using Dbl-Het reporter mice (*n* = 6/group), macrophages (CD11b^+^F4/80^+^) were delineated based upon their relative expression of GFP (*CX3CR1*) from RFP (*CCR2*) by flow cytometry. There were relatively very few CCR2^+^ macrophages (*blue box*) in the sham animals; however, there was a significant increase in this subpopulation due to age. However, 24 h following TBI, there was a significant increase in the mean number of CCR2^+^ macrophages (*blue box*) in the aged animals compared to young. Data were analyzed using Student’s *t* test and are represented by the mean + SEM. **p < 0.01

Comparatively, we examined if these gene expression responses were predictive of downstream disease-based functions using IPA software. Using IPA comparative analysis, we next sorted the top ten biological functions by the activation *z*-score in the aged TBI group. Of the top ten functions generated, there was a general theme of each related to innate immune response. In particular, there was an overrepresentation with both the differentiation and activation of monocytes in the aged TBI group, relative to young TBI (Fig. 4a; arrow). Examination of the putative response network associated with the activation of monocytes (Fig. 4a) revealed significant differential expression patterns in the mediators affecting this response system (Fig. 4b). Principal to this response was the induction of pro-inflammatory and chemotactic mediators within the CC and CXC motifs.

### Age exaggerates the accumulation of CCR2^+^ monocytes following trauma

We next sought to determine if the predictive responses generated in IPA translated to in vivo reactions. Using the Dbl-Het reporter mice, our data show that aging results in a small, but significant increase in the mean number of CCR2^+^ infiltrated monocytes/macrophages relative to young mice (Fig. 4c). When compared to this result, following brain injury, there is a much greater recruitment and infiltration of CD11b^+^F4/80^+^CCR2^+^ macrophages into the parenchyma as compared to young animals. The nature of these cells in the diseased brain remains controversial, as there are reports detailing both beneficial and detrimental actions of this population of monocytes varying among disease models [13, 24–26]. Interestingly, as a response of age, there was a visual increase in the number of resident F4/80^+^ microglia/macrophages (CX3CR1^+^CCR2^−^; Fig. 4c), which was exaggerated in response to TBI. Recent work has shown that this subpopulation may have distinct roles in the inflammatory response to trauma [27].

### Simultaneous CCR2/5 antagonism abrogates age-related response to injury

**Fig. 5:**
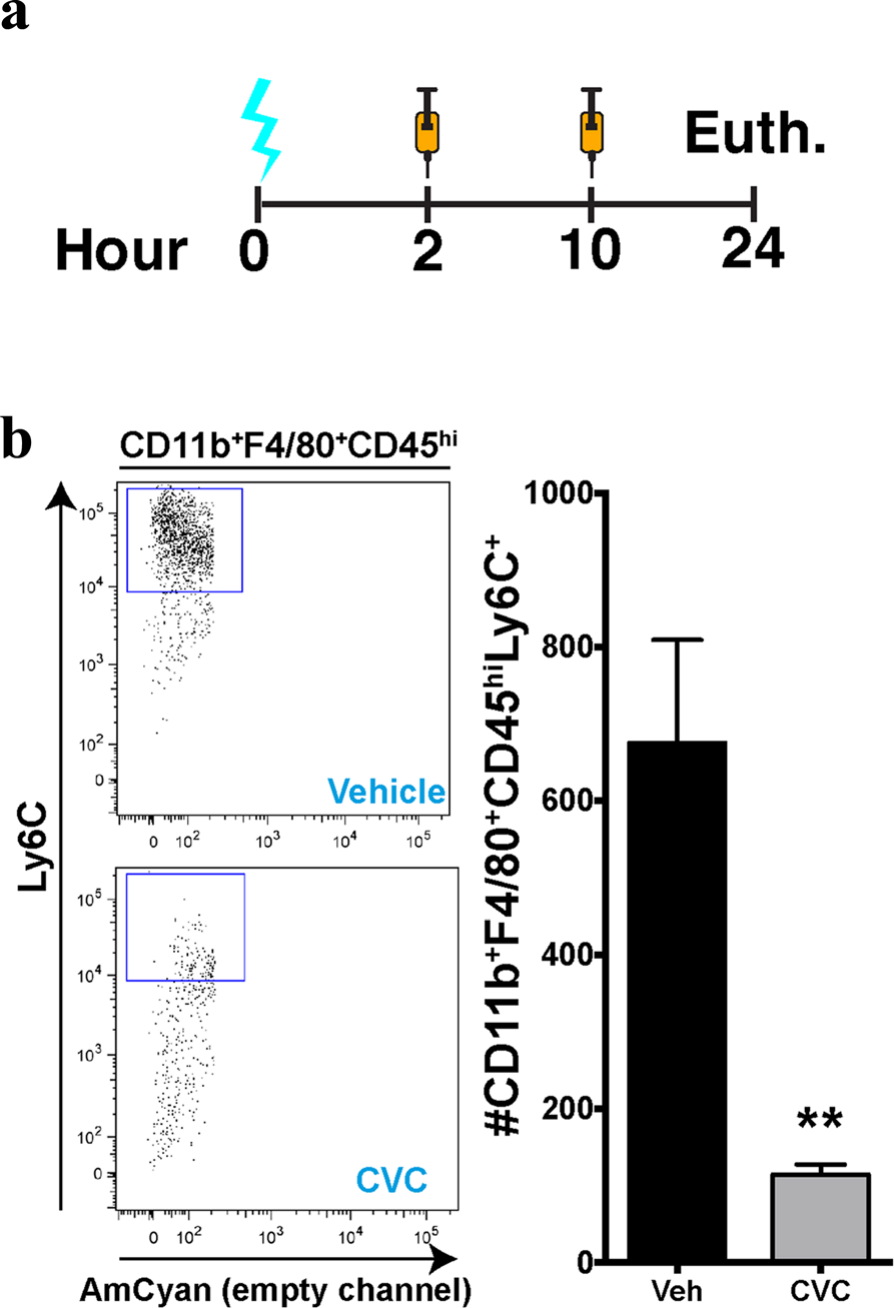
Dual targeting of CCR2/5 with CVC mitigates TBI-induced macrophage recruitment. **a** In WT mice, CVC or vehicle was administered BID via oral gavage at 100 mg/kg at 2 and 10 h post surgery before animals were euthanized for various endpoints at 24 h following surgery. **b** A cohort of WT aged TBI animals (*n* = 8/group) was used for flow cytometry analysis of macrophage infiltration into the injured brain. Twenty-four hours after injury, there was a significant decrease in the number of peripheral macrophages (CD11b^+^F4/80^+^CD45^hi^Ly6C^+^) in the CVC-treated animals compared to their vehicle-treated counterparts

In order to abrogate this response, we treated aged TBI mice with cenicriviroc (CVC), a potent, oral, dual-antagonist of CCR2/5, which is currently being evaluated in a phase 2 clinical trial in adults with non-alcoholic fatty liver disease and liver fibrosis (NCT02217475). Separate cohorts of aged TBI animals were treated with either vehicle or CVC twice daily several hours after injury (Fig. 5a). Using this experimental approach, we observed a significant reduction in the numbers of CD11b^+^F4/80^+^CD45^hi^Ly6C^+^ macrophages (Fig. 5b,c), which are analogous to CCR2^+^ macrophages, recruited into the diseased brain [12, 28].

### CVC treatment mitigates the age-related inflammatory response to TBI

**Fig. 6:**
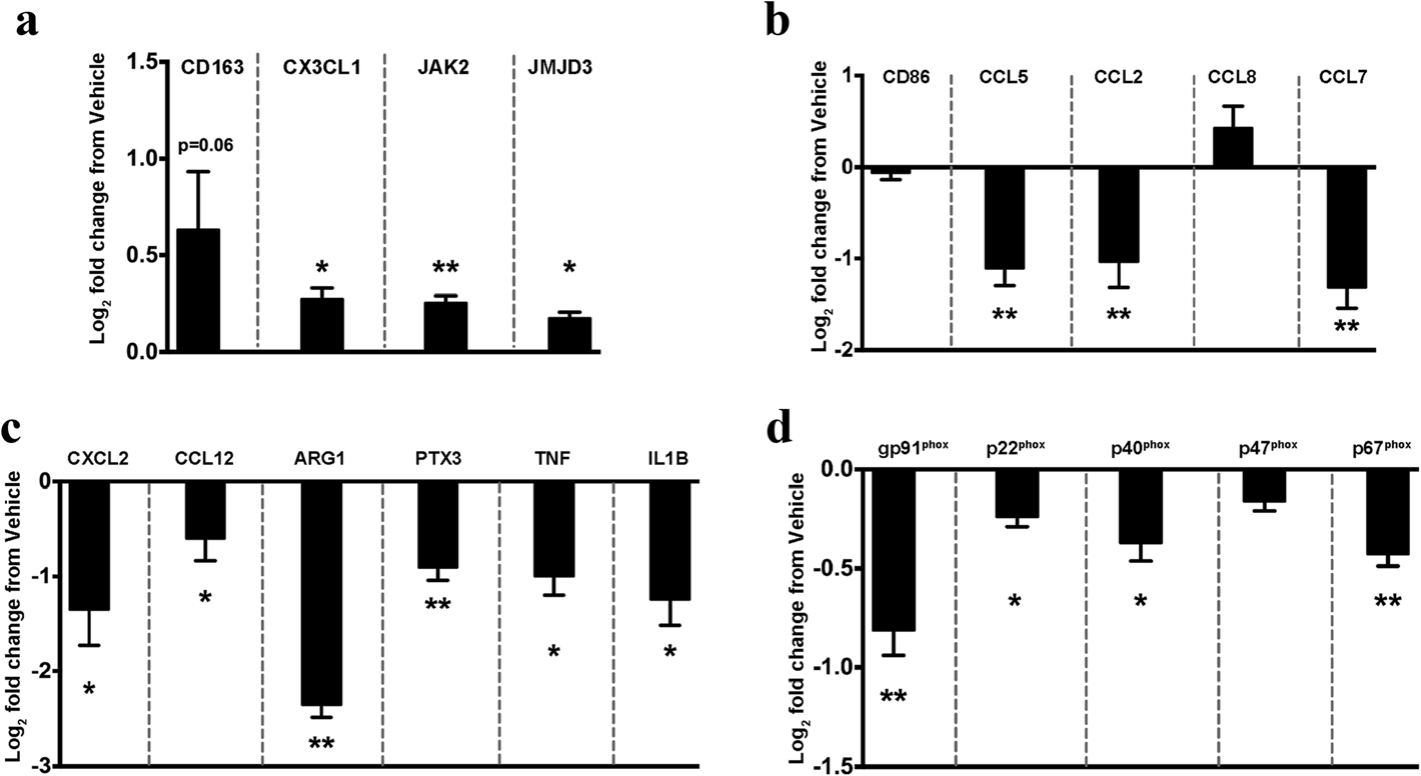
Treatment with CVC attenuates age-related inflammatory and oxidative stress responses. **a-c** In a separate cohort (*n* = 8/group), inflammatory clusters were examined for their response to CVC treatment. **a**–**c** Correspond to the previously examined green, red, and aquamarine expression clusters, respectively. **d** Subunits of the NOX2 complex were measured as a response to CVC treatment (*n* = 8/group). Gene expression data are relative to vehicle-treated aged TBI. Vehicle expression values are equivalent to zero. Data were analyzed using Student’s *t* test are represented by mean + SEM. *p < 0.05, **p < 0.01, and ***p < 0.001

We next examined whether this treatment had any effect within the previously defined (Fig. 1) expression patterns identified by hierarchical clustering. Treatment with CVC significantly increased the expression of the genes in the green “restraint and anti-inflammatory” cluster (Fig. 6a), while significantly downregulating genes from the red “inflammatory chemotactic” cluster (Fig. 6b) and aquamarine “injury-induced inflammation” (Fig. 6c) gene clusters. The putative upstream network analysis predicted a proclivity for exaggerated oxidative stress response in the aged TBI group through downregulation of GPX1-regulated pathways (Fig. 3c). Importantly, we have recently shown that CCR2 antagonism ameliorates constituents mediating oxidative stress response following TBI through reduction of NADPH oxidase (NOX2) complex [13]. In agreement with those findings, our current data show that CVC treatment significantly downregulated multiple constituents of the neurotoxic ROS complex, NOX2 (Fig. 6d), which were previously shown to be upregulated in aged animals following TBI [22].

## Discussion

The brain’s response to neurotrauma initiates a wide range of molecular signaling mechanisms involving a variety of cell types [29, 30]. Of principle concern is the activation of the myeloid constituents, microglia, and monocytes, due to their involvement and propagation of a variety of acute and chronic disorders [31]. The approaches taken in this study define the brain’s inflammatory milieu in response to TBI and how these molecular patterns are altered as a function of age. The altered shift in expression patterns of chemotactic mediators in aged TBI brain created a permissive environment for the exaggerated influx of peripheral monocytes/macrophages to the brain. This, in part, may be due to redundant increases in chemokines associated with CCR2/5 signaling. While the role of these monocyte populations is diverse across various neurological diseases, we have shown that these cells augment a pro-inflammatory and potentially neurotoxic bias in the aged brain. We also used an available CCR2/5 inhibitor to reduce the accumulation of pro-inflammatory myeloid cells in the brain. Cumulatively, our findings demonstrate that the CCR2-mediated recruitment of monocytes into the injured brain is a rational therapeutic target that may abrogate TBI-induced neuroinflammatory-mediated sequelae.

We recently described the leukocyte-mediated neuroinflammatory sequelae associated with this TBI model in young animals over a detailed time course spanning acute through chronic time points [13]. Our previous findings suggest that resident microglia are responsible for the initiation of specific inflammatory mediators and infiltrated CCR2^+^ macrophages significantly augment this response. However, in the context of aging, recent work has shown that the aged brain has an altered inflammatory profile relative to basal levels of adult mice [32], potentially priming these systems for maladaptive responses to insult or injury [33]. In the context of neurotrauma, our current data suggest that age dysregulates multiple signaling networks associated with the activation, chemotaxis, and inflammatory response of innate immune effectors. We have recently shown that these responses do not fall within the dichotomous constraints of “M1/M2” polarization [15]. In agreement with or previous work, our current data indicate that as a result of age, there is dysregulation across both pro- and anti-inflammatory mediators. While we highlighted several instances of exaggerated pro-inflammatory and chemotactic gene profiles using unsupervised hierarchical clustering, we also observed pro-inflammatory mediators such as CD40, CXCL11, CCL17, CCL19, and IL16 that were downregulated in the aged TBI group, relative to young TBI. Given these opposing inflammatory responses, it is difficult to conclude that TBI in the aged predisposes a purely pro-inflammatory bias, as there are both inductions of anti-inflammatory response with concomitant downregulation of pro-inflammatory responses, relative to young injured animals. However, our current data attribute, in part, that cumulatively, these maladaptive responses create an overly permissive environment for the exaggerated recruitment and accumulation of CCR2^+^ macrophages.

Although CCR2 is expressed on a variety of immune cells, circulating “inflammatory” monocytes are the main population that expresses this cytokine receptor [12]. CCR2 is currently known to have five cognate ligands; CCL2, CCL8, CCL7, CCL13 (human only), and CCL12 [34, 35]. However, there is a degree of promiscuity of the CCR2 receptor and its ligands as they are known to bind other C-C receptors; notably CCR5 [36, 37]. Expression of CCR5, like CCR2, is found on a variety of circulating immune effectors, but most relevant to our study, is co-expressed on CCR2^+^ monocytes [38]. Overall, in the context of our current findings, the cross-talk and co-expression of CCR2/5 on monocytes, and enhanced expression of their cognate ligands may potentiate the increased recruitment we observed in aged TBI animals. Therefore, our current data are consistent with the formation of a pro-chemotactic milieu created, in part, by increased signaling through CCR2/5 expressing cells.

NOX2 activation possesses a neurotoxic nature from the production of ROS intermediates [39, 40] as well as maintaining redox pro-inflammatory signaling cascades of macrophages [41]. Recent work has detailed an increase in some subunits of the ROS-producing NOX2 complex of aged TBI animals [22]. Our treatment paradigm with CVC significantly downregulated the expression of the multiple subunits that comprise the NOX2 complex, suggesting a decreased propensity for ROS intermediate formation relative to the vehicle-treated animals. These data align with our previous work implicating CCR2^+^ macrophages as the predominant source for NOX2 gene expression following TBI [13]. Importantly, in rodent models of TBI, inhibiting this complex decreased neuronal damage and improved recovery [42, 43].

## Conclusions

Our current study demonstrates that the aged brain’s response to neurotrauma involves the exacerbated expression of multiple mediators involved in the recruitment of peripheral macrophages to the injured parenchyma. Blocking of these innate immune mediators via CCR2/5 antagonism significantly blunted their ingress, while concomitantly reducing inflammatory and potentially neurotoxic signatures. Intentionally, we limited the scope of this study to examine the period following injury where the maximum inflection of recruited CCR2^+^ macrophages was previously known [13], in an effort to define this time point within the context of age at the time of TBI. Therefore, the conclusions garnered in this study should not be expanded beyond the presented injury paradigm. Future work is needed to define how neurotrauma in the context of age affects inflammatory response in subacute and chronic periods and whether the responses and treatment currently examined may produce tangible outcome measures in those periods.
